# Local Anesthetic Systemic Toxicity Following High-Volume Ropivacaine, Epinephrine, Clonidine, and Ketorolac (RECK) Infiltration During Total Knee Arthroplasty: A Case Report

**DOI:** 10.7759/cureus.116865

**Published:** 2026-09-24

**Authors:** Brian Mendelson, Jose Perdomo Trejo, Soliana Bekele

**Affiliations:** 1 Anesthesia, Duke University, Durham, USA

**Keywords:** lipid emulsion, local anaesthetic systemic toxicity, local infiltration analgesia, ropivacaine, total knee arthroplasty

## Abstract

Local infiltration analgesia is widely used for post-operative pain control following joint arthroplasty and is generally considered to carry a low risk of local anesthetic systemic toxicity. We report a case of local anesthetic systemic toxicity in a 72-year-old woman following primary total knee arthroplasty performed under spinal anesthesia with adjunctive adductor canal block and high-volume periarticular infiltration with ropivacaine, epinephrine, clonidine, and ketorolac (RECK) solution. The cumulative perioperative ropivacaine dose was 286 mg (3.9 mg/kg). Soon after arrival in the post-anesthesia care unit, the patient developed acute confusion and focal myoclonic activity in the right arm without cardiovascular instability. Lipid emulsion was administered with rapid and complete neurological recovery. This case highlights the importance of considering cumulative dosing across regional and surgical infiltration techniques, the influence of patient-specific factors on susceptibility to toxicity, and the need for vigilance for delayed manifestations of local anesthetic systemic toxicity following high-volume infiltration.

## Introduction

Local infiltration analgesia (LIA) is widely used for postoperative pain control following joint arthroplasty [1,2]. The technique typically involves administration of dilute local anesthetic, with or without adjuncts such as epinephrine and ketorolac. Although LIA commonly involves large absolute doses of local anesthetic, reports of local anesthetic systemic toxicity (LAST) associated with this technique remain uncommon. We describe a case of LAST following high-volume periarticular infiltration with ropivacaine, epinephrine, clonidine, and ketorolac (RECK) solution, a combination that is increasingly used given evidence of beneficial effects on postoperative pain control [3,4]. To our knowledge, this is the first reported case of LAST attributable to RECK infiltration. Furthermore, the timing of the seizure upon arrival in the recovery area is unusual. This report highlights considerations regarding cumulative dosing across techniques, patient-specific risk factors, and the need for vigilance for delayed manifestations of LAST.

## Case presentation

A 72-year-old, 74-kg woman (American Society of Anesthesiologists physical status II) presented for primary total knee arthroplasty. Her medical history included well-controlled asthma, hypertension, and gastroesophageal reflux disease. Spinal anesthesia was performed with 100 mg lidocaine, and an adductor canal block was placed with 40 mg ropivacaine in 1:400,000 epinephrine. Sedation during the procedure was maintained with a propofol infusion at a dose of 70 micrograms per kilogram per minute for the majority of the case, which lasted for 84 minutes. The intra-operative course was otherwise unremarkable; she received antibiotic prophylaxis with cefazolin and a total of 1.5 g tranexamic acid. A pneumatic tourniquet was not utilized. Intra-operatively, the surgical team performed periarticular infiltration with 100 mL of RECK solution containing 246 mg ropivacaine, 0.5 mg epinephrine, 80 µg clonidine, and 15 mg ketorolac. The cumulative perioperative ropivacaine dose was 286 mg (3.9 mg/kg). This solution is commonly used during total knee arthroplasty and has been shown to help with postoperative analgesia.

On arrival in the post-anesthesia care unit, she was initially stable and neurologically intact, moving all four limbs and conversant. Soon after arrival, she became disoriented and unresponsive to voice and developed focal myoclonic jerks in the right arm. Cardiovascular parameters remained stable throughout. Given the cumulative ropivacaine dose, the absence of any preceding medication that could account for her presentation, and no history of thromboembolic disease, LAST was the leading diagnosis. Lipid emulsion was administered as a 100 mL bolus followed by an infusion of 250 mL, consistent with guidelines from the American Society of Regional Anesthesia and Pain Medicine, with return to baseline neurological status within minutes. She was monitored in the recovery area for a further 2 hours with no recurrence of symptoms. 

## Discussion

High-volume LIA is often presumed to be safe because of gradual systemic absorption from the surgical site. Studies characterizing plasma concentrations of ropivacaine following high-volume periarticular infiltration in lower limb arthroplasty have demonstrated that peak systemic levels typically remain below established toxic thresholds, particularly for the unbound fraction [5]. In this case, the cumulative perioperative ropivacaine dose (286 mg, comprising an adductor canal block and periarticular infiltration) exceeded the commonly cited weight-based ceiling of approximately 3 mg/kg [6]. Toxicity, however, is unlikely to be determined by absolute dose alone; absorption kinetics, plasma protein binding and patient-specific factors are also critical determinants [6]. Miller et al. demonstrated that female sex, advanced age, body habitus and renal function predict peak plasma ropivacaine concentration following high-volume LIA, and that periarticular injection into the knee joint produces a delayed time-to-peak compared with perineural techniques and infiltration at other sites [5]. Collectively, these observations reinforce the importance of an individualized approach to local anesthetic administration that accounts for patient characteristics, procedural factors, and cumulative dose.

Advanced age likely contributed to this patient’s susceptibility to LAST. Older patients have reduced hepatic blood flow and diminished clearance of amide local anesthetics, resulting in higher and more prolonged plasma concentrations [7,8]. Age-related changes in central nervous system sensitivity may also lower the threshold for neurological toxicity [9]. Reliance on total body weight for dose calculation can overestimate the safe dose in older patients with reduced lean body mass [7,8]. Although designated as American Society of Anesthesiologists (ASA) physical status II, this classification may not fully reflect the physiological vulnerability associated with advanced age. 

The clinical presentation in this case was notable for predominantly neurological manifestations - acute confusion and focal myoclonic activity - without cardiovascular instability. Early recognition and prompt administration of lipid emulsion therapy resulted in rapid and complete neurological recovery. American Society of Regional Anesthesia and Pain Medicine (ASRA) guidelines emphasize early lipid therapy at the first signs of suspected LAST, even in the absence of cardiovascular compromise [10], to prevent progression to severe neurological or cardiac toxicity. The delayed onset of symptoms after arrival in the post-anesthesia care unit underscores the importance of continued postoperative vigilance following high-volume or cumulative local anesthetic administration. We considered that the intra-operative propofol infusion may have masked or suppressed early neurological signs of toxicity, with breakthrough symptoms becoming apparent only after the infusion was discontinued and the patient transferred to the recovery area.

This case has several implications for clinical practice. There is a clear need for explicit preoperative communication between anesthesia and surgical teams regarding cumulative local anesthetic dosing, including agreement on a safe ceiling dose and consideration of whether the planned regional and infiltration techniques can both be performed within that ceiling. Where a regional block has been performed, reduction in the volume or concentration of subsequent surgical infiltration should be considered. Conversely, if the surgical infiltration dose cannot be reduced, anesthetists may need to reconsider whether to perform a peripheral nerve block at all. 

The procedural context of administration also differs between regional and surgical techniques. Anesthetists typically inject local anesthetic in fractionated 3-5 mL aliquots under continuous hemodynamic monitoring, which facilitates early detection of inadvertent intravascular injection. Surgical periarticular infiltration is generally performed as a more continuous injection without routine aspiration or accompanying hemodynamic surveillance, unless the anesthesia team is alerted that injection is taking place. The use of a pneumatic tourniquet, common in lower limb arthroplasty, may further confound assessment: arterial occlusion can both delay systemic absorption and produce false-negative aspirations during injection, potentially contributing to delayed presentation of toxicity once the tourniquet is released. To our knowledge, no published studies have directly compared the risk of LAST between anesthetist-performed adductor canal block and surgeon-performed LIA, although the latter typically involves several-fold more local anesthetic. Careful patient selection and risk stratification are essential when planning high-volume LIA, particularly in patients at higher risk or those receiving additional regional techniques. Risk-mitigation strategies include dose reduction, use of ideal or lean body weight for dose calculation, implementation of institutional ceiling doses, and a heightened threshold for prolonged postoperative monitoring after high-volume infiltration.

## Conclusions

In conclusion, this case challenges the assumption that high-volume LIA is uniformly safe and underscores the need for vigilance for delayed manifestations of LAST throughout the perioperative period. As LIA techniques become more widely adopted, further investigation into the pharmacokinetics of large-volume local anaesthetic infiltration, particularly in older and higher-risk patients, is warranted to refine dosing guidance and improve patient safety.
